# Supplementary material for: Gold-Nanoparticle-Coated Magnetic Beads for ALP-Enzyme-Based Electrochemical Immunosensing in Human Plasma
Source: Materials (Basel). 2022 Oct 3;15(19):6875. doi: 10.3390/ma15196875 (PMC9573121; doi:10.3390/ma15196875)
Supplement: Supplementary file 1 [file materials-15-06875-s001.zip › materials-1900011-supplementary.pdf]

materials

MDPI

*Supplementary Material*

# Gold-Nanoparticle-Coated Magnetic Beads for ALP-Enzyme-Based Electrochemical Immunosensing in Human Plasma

**Seo-Eun Lee [1,2,†], Se-Eun Jeong [1,2,†], Jae-Sang Hong [3], Hyungsoon Im [3,4], Sei-Young Hwang [1], Jun Kyun Oh [2] and Seong-Eun Kim [1,*]**

[1] Human IT Convergence Research Center, Convergence System R&D Division, Korea Electronics Technology Institute (KETI)), 25 Saenari-ro, Bundang-gu, Seongnam-si, Gyeonggi-do 13509, Korea
[2] Department of Polymer Science and Engineering, Dankook University, 152 Jukjeon-ro, Suji-gu, Yongin-si, Gyeonggi-do 16890, Korea
[3] Center for Systems Biology, Massachusetts General Hospital, Boston, MA 02114, USA
[4] Department of Radiology, Massachusetts General Hospital, Boston, MA 02114, USA
* Correspondence: sekim@keti.re.kr; Tel.: +82-31-789-7555
† These authors contributed equally to this work.

## Supplementary information

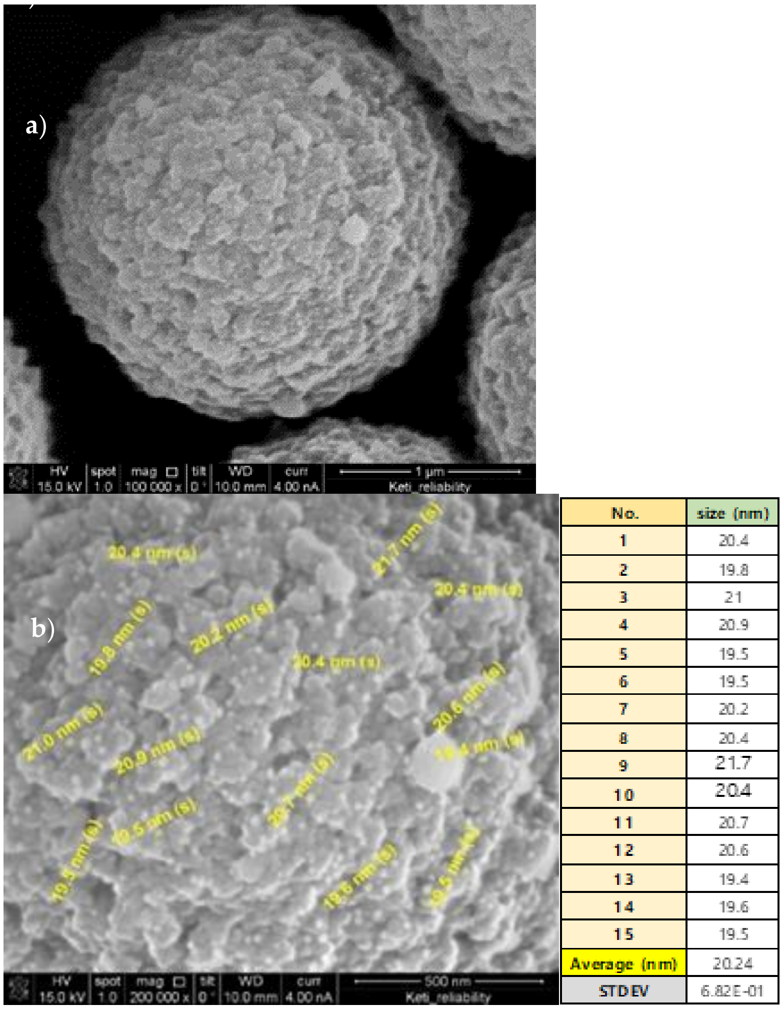


| No. | size (nm) |
|---|---|
| 1 | 20.4 |
| 2 | 19.8 |
| 3 | 21 |
| 4 | 20.9 |
| 5 | 19.5 |
| 6 | 19.5 |
| 7 | 20.2 |
| 8 | 20.4 |
| 9 | 21.7 |
| 10 | 20.4 |
| 11 | 20.7 |
| 12 | 20.6 |
| 13 | 19.4 |
| 14 | 19.6 |
| 15 | 19.5 |
| Average (nm) | 20.24 |
| STDEV | 6.82E-01 |

**Figure S1.** SEM images of AuNP-MB (AMB): (**a**) 100,000×; (**b**) 200,000× and analysis of AuNP size (n = 15).

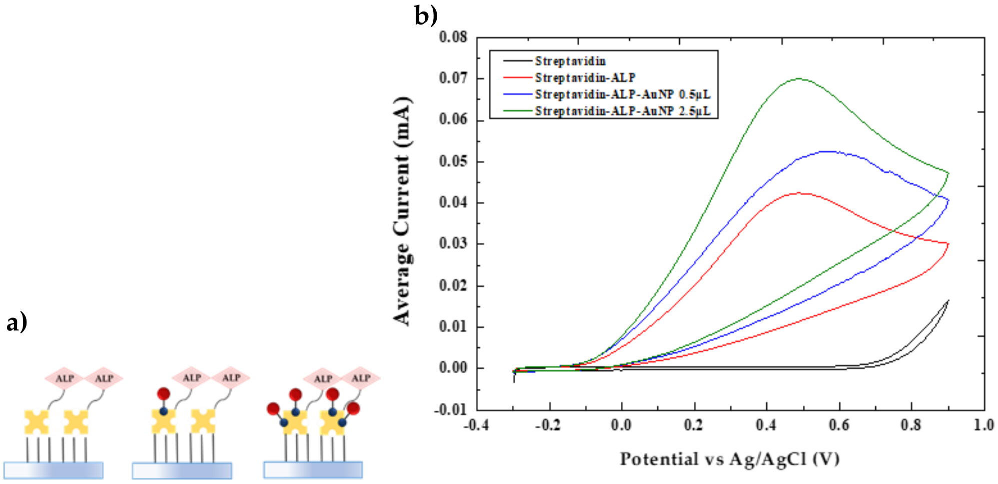


**Figure S2.** Analysis of EC signal enhancement of ALP with AuNP on ITO electrode: (**a**) Schematics for streptavidin labeled with ALP immobilized on ITO electrode treated with various amount of biotinylated AuNP (yellow: streptavidin; red: AuNP; black: biotin); (**b**) Cyclic voltammetry (ranging from −0.3 V to 0.9 V) results of ALPs with various amount of AuNP on ITO electrode.

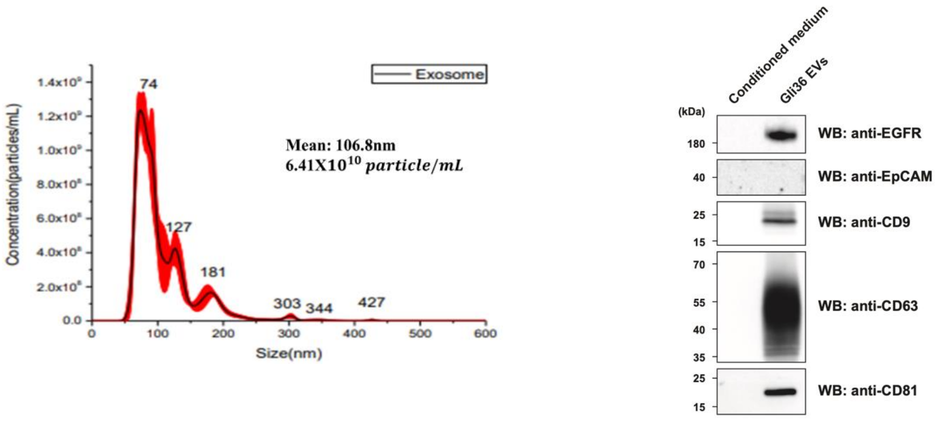


(**a**) (**b**)

**Figure S3.** EV characterization. **(a)** Nanoparticle tracking analysis (NTA) quantification of Gli36 GBM-derived EV. NTA distribution of the EVs and concentration (particle/mL) of EVs. Mean of EV diameter (n=3) is shown in the graph. **(b)** Western blot analysis of Gli36 EVs and culture medium. Strong signals for tetraspanins (CD9, CD63, CD81) and EGFR were observed in EVs from the Gli36 cell line while no signal was detected for EpCAM.

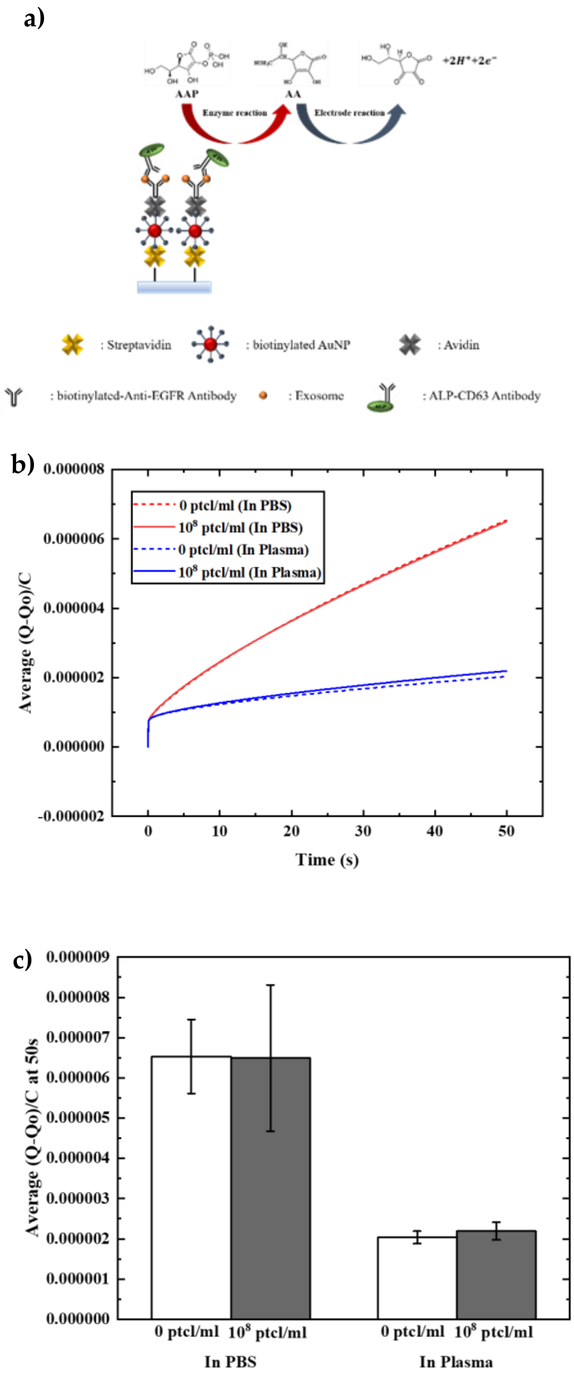


**Figure S4.** Schematics for AuNP used platform on ITO electrode without MB for EV detection (**a**) and chronocoulogram recorded (at 0.6 V); (**b**) and charge values at 50 sec from SI Fig.4b were shown at 50 sec after an incubation period of 2 min at immunosensing electrodes treated with different concentrations of EV and anti- EGFR antibodies in PBS or in 90% of human plasma; (**c**); Data = mean ± standard deviation, n = 4.
